# Analysis of sagittal profile and radiographic parameters in symptomatic thoracolumbar disc herniation patients

**DOI:** 10.1186/s12891-021-04033-x

**Published:** 2021-02-12

**Authors:** Ang Gao, Yongqiang Wang, Miao Yu, Xiaoguang Liu

**Affiliations:** Department of Orthopaedics, Peking University Third Hospital, No. 49 North Garden Road, Haidian District, Beijing, 100191 China

**Keywords:** Thoracolumbar disc herniation, Sagittal alignment, Spinopelvic parameters, Roussouly classification, Thoracolumbar kyphosis

## Abstract

**Background:**

Few studies describe thoracolumbar disc herniation (TLDH) as an isolated category, it is frequently classified as the lower thoracic spine or upper lumbar spine. Thus, less is known about the morphology and aetiology of TLDH compared to lumbar disc herniation (LDH). The aim of study is to investigate sagittal alignment in TLDH and analyze sagittal profile with radiographic parameters.

**Methods:**

Data from 70 patients diagnosed with TLDH were retrospectively reviewed. The thoracic-lumbar alignment was depicted by description of curvatures (the apex of lumbar curvature, the apex of thoracic curvature, and inflexion point of the two curvatures) and radiographic parameters from complete standing long-cassette spine radiographs. The rank sum test was utilised to compare radiographic parameter values in each subtype.

**Results:**

We found two subtypes differentiated by the apex of thoracic kyphotic curves. The sagittal profile was similar to that of the normal population in type I, presenting the apex of the thoracic kyphotic curve located in the middle thoracic spine. The well aligned thoracic-lumbar curve was disrupted in type II, presenting the apex of the thoracic kyphotic curve located in the thoracolumbar region in type II patients. Thirty-six patients were classified as type I, and 34 patients were classified as type II. The mean sagittal vertical axis, T1 pelvic angle and L1 pelvic angle were 27.9 ± 24.8°, 8.2 ± 7.3° and 6.2 ± 4.9°, respectively. There was significant difference (*p* < 0.001) of thoracolumbar angle between type I (14.9 ± 7.9°) and type II patients (29.1 ± 13.7°).

**Conclusions:**

We presented two distinctive sagittal profiles in TLDH patients, and a regional kyphotic deformity with a balanced spine was validated in both subtypes. In type I patients, disc degeneration was accelerated by regional kyphosis in the thoracolumbar junction and eventually caused disc herniation. In type II patients, excessive mechanical stress was directly loaded at the top of the curve (thoracolumbar apex region) rather than being diverted by an arc as in a normal population or type I patients. Mismatch between shape and sacral slope value was observed, and better agreement was found in Type II patients.

## Background

In normal spine alignment, the thoracolumbar spine is routinely defined as T10-L2 and recognized as the inflexion region between the kyphotic thoracic spine and lordotic lumbar spine [1]. Although few studies describe it as an isolated category, it is frequently classified as the lower thoracic spine or upper lumbar spine in many studies [2, 3]. Thus, less is known about the morphology and aetiology of thoracolumbar disc herniation (TLDH) compared to lumbar disc herniation (LDH) [1].

It is widely accepted that explicit descriptions of a sagittal spine alignment assist in the determination of aetiology and the orientation of the surgical plan, both of which are achieved using two incisive tools (spinopelvic parameters and curvatures) in both degenerative spine disease and spine deformity [4–6]. However, these descriptions are overemphasized on spinopelvic parameters, and this process is sometimes tedious due to the lack of a direct view of spine curvatures. Furthermore, the sagittal profile may be affected by complex compensatory mechanisms, especially in patients with a spine deformity, which may not be indicated in radiographic parameters [6]. To address these deficiencies, Albelin-Genovois et al. described sagittal alignment with a direct view of spine curvatures and supplemented this with relevant spinopelvic parameters, which were validated in a further study [6, 7].

We agreed with the Albelin-Genovois strategy in that an ideal and practical description of spine alignment should be based on a direct view of spine curvatures and supplemented with radiographic parameters [6]. Guided by this “Curvature First” tactic, regional curvatures of the thoracic and lumbar spine were first recorded separately and then pieced together to depict a direct view of the whole thoracic and lumbar spine in this study. Here, we present some exclusive sagittal alignment descriptions of TLDH, investigate the association between TLDH and curvature variants, and interpret alignment descriptions with spinopelvic parameters in this relatively rare condition.

## Methods

### Compliance with ethical standards

All procedures performed in studies involving human participants were in accordance with the ethical standards of the institutional and/or national research committee and with the 1964 Helsinki declaration and its later amendments or comparable ethical standards. For this type of study (retrospective study), formal consent was not required.

### Inclusion criteria

Our hospital’s electronic database was retrospectively reviewed. Patients seen from January 2013 to December 2019 who met the following criteria were included: (1) age > 18 years; (2) symptomatic disc herniation at T10/T11, T11/T12, T12/L1, and L1/L2 level(s); and (3) availability of complete standing long-cassette anteroposterior and lateral spine radiographs.

### Exclusion criteria

The exclusion criteria were as follows: (1) concomitant coronal spine deformity (Cobb angle > 10°); (2) concomitant infection, spine fracture, or tumour; (3) previous spine or hip surgery; and (4) neuromuscular spinal abnormalities.

### Patient demographics and description of sagittal thoracolumbar profile

The patients’ demographic data, including age, sex, body mass index (BMI), history of smoking, history of alcohol consumption, and diabetes mellitus (DM) history were extracted from our electronic medical database.

The thoracic-lumbar alignment was depicted in two steps: (1) description of curvatures and (2) supplementary radiographic parameters.

Curvatures were depicted by three variants: (1) the apex of lumbar curvature, (2) the apex of thoracic curvature, and (3) inflexion point of the two curvatures. The association between TLDH level and curvature variants (apex and inflexion point) was also recorded (e.g.: TLDH at the apex region [TLDH-A], TLDH at the inflexion region [TLDH- I], and THDH at other regions [TLDH-O]).

Radiographic parameters, including coronal vertical axis (CVA), sagittal vertical axis (SVA), pelvic incidence (PI), sacral slope (SS), lumbar lordosis (LL), PI-LL, thoracic kyphosis (TK), thoracolumbar kyphosis (TLK), T1 pelvic angle (T1PA), and L1 pelvic angle (L1PA) were obtained from the anteroposterior and lateral X-rays. All parameters were measured separately by two expert spine surgeons.

LL was defined as the sagittal Cobb angle from the superior end plate of L1 to the sacral end plate. TK was defined as the sagittal Cobb angle from the superior end plate of T4 to the T12 lower end plate. TLK was defined as the sagittal Cobb angle from the superior end plate of T11 to the L2 lower end plate. T1PA was defined as the angle between the line from the femoral head axis to the centroid of T1 and the line from the femoral head axis to the middle of the superior endplate of S1 [8]. L1PA was defined as the angle formed by a line from the centre of the L1 vertebral body to the femoral head axis and a line from the femoral head axis to the centre of the S1 endplate [9].

The Roussouly classification was identified as the apex of lumbar lordosis and the SS value, and these two variants were well-matched in each subtype in the normal population [10]. We set the apex of lumbar lordosis as the ideal subtype and investigated whether the corresponding SS value was matched according to the Roussouly classification.

### Statistical analysis

Clinical and radiographic data were analysed using the Statistical Package for the Social Sciences version 22.0 (SPSS, Inc., Chicago, IL, USA). The inter-rater reliability of the classification was tested by interclass correlation coefficients (ICCs). The rank-sum test and chi-square test were utilised to compare patient demographics and radiographic parameter values in each subtype, and the Fisher’s exact test was used to compare the differences of TLDH-A and TLDH- I occurrence and concordance with the Roussouly classification in each subtype. Furthermore, *p* values < 0.05 were considered statistically significant.

## Results

### Patient characteristics

Between January 2013 and December 2019, 70 patients (53 men, 17 women) with a mean age of 45.5 ± 1.7 (range, 19–73) years and BMI of 26.5 ± 4.3 (range, 19.9–38.0) were enrolled in this study. Among this population, six patients had a history of DM, eight patients had a history of smoking, and eight reported a history of alcohol consumption.

One level of TLHD was validated in 58 patients, two levels in 10 patients, and three levels in two patients. The distributions of levels were as follows: 12 cases, T10/T11; 34 cases, T11/T12; 24 cases, T12/L1; and 14 cases, L1/2.

The distribution of each radiographic parameter and reliability of the radiographic parameters are presented in Table 1.

**Table 1:** The values and inter observer reliability for radiographic parameter

Parameters	Mean	Standard deviation	Range	Inter-rater ICC
SVA(mm)	27.9	24.8	−46.9-73.1	0.90
CVA(mm)	7.9	5.3	2.1–26.1	0.92
TK(°)	29.1	12.3	0.8–56.0	0.91
TLK(°)	21.8	13.1	0.9–57.0	0.88
LL(°)	47.5	14.9	7.5–71.4	0.84
T1PA(°)	8.2	7.3	0.3–43.3	0.91
L1PA(°)	6.2	4.9	0.2–19.6	0.89
PI(°)	45.9	7.5	30.7–63.6	0.86
SS(°)	34.6	7.9	14.1–53.1	0.92
PT(°)	11.4	9.4	0.2–38.3	0.90
PI-LL(°)	12.4	9.4	0.5–44.9	0.88

### Subtypes of sagittal thoracic-lumbar alignment

**Fig. 1:**
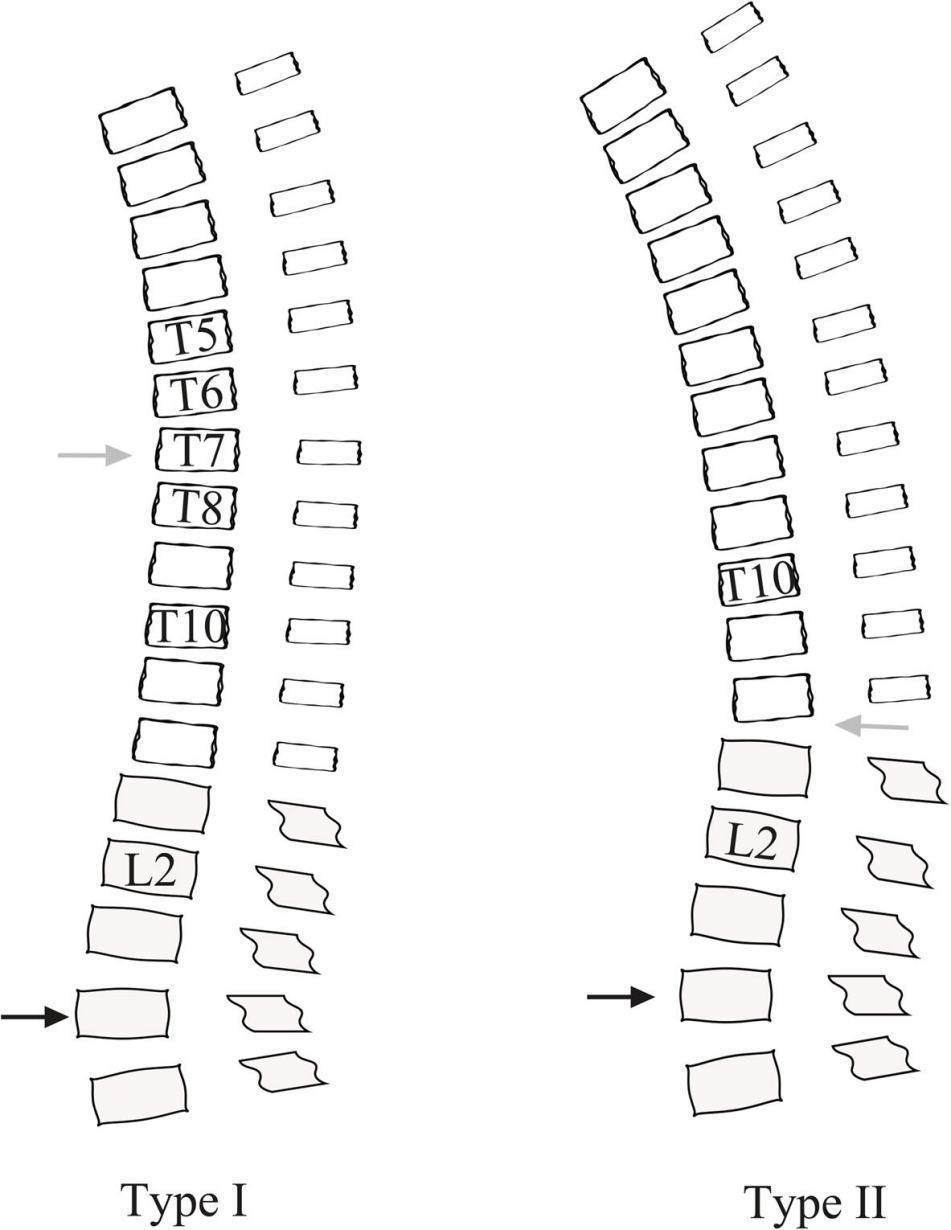
Illustration of two subtypes. In type I patients, grey arrow demonstrated the apex of thoracic kyphotic curve, which was located at middle thoracic region (T5-T8); and black arrow demonstrated the apex of lumbar lordotic curve. In type II patients, grey arrow demonstrated the apex of thoracic kyphotic curve, which was located at thoracolumbar region (T10-L2); and black arrow demonstrated the apex of lumbar lordotic curve

**Fig. 2:**
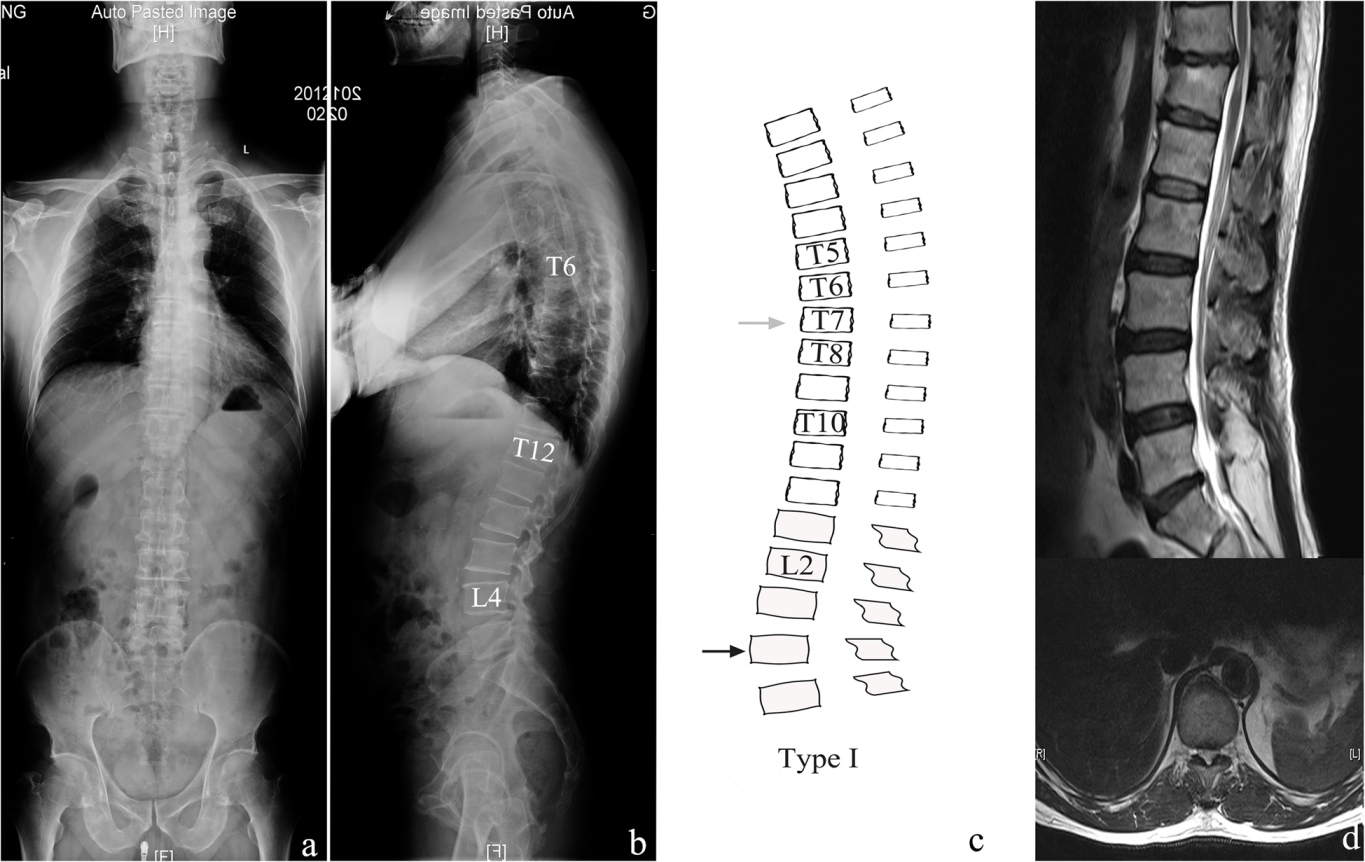
A 52-year-old man classified as type I. **a** Anterior-posterior whole spine x-ray demonstrated a balanced spine in coronal alignment. **b** Apex of lumbar lordotic curve, apex of thoracic kyphotic curve, and inflexion point of two curves was identified as L4, T6, and T12, respectively. A balanced spine in sagittal alignment was also observed in lateral x-ray. **c** Illustration of type I, and it had good agreement to a real case. **d** MR demonstrated disc herniation at T11/T12 level, and a TLDH-I was identified

**Fig. 3:**
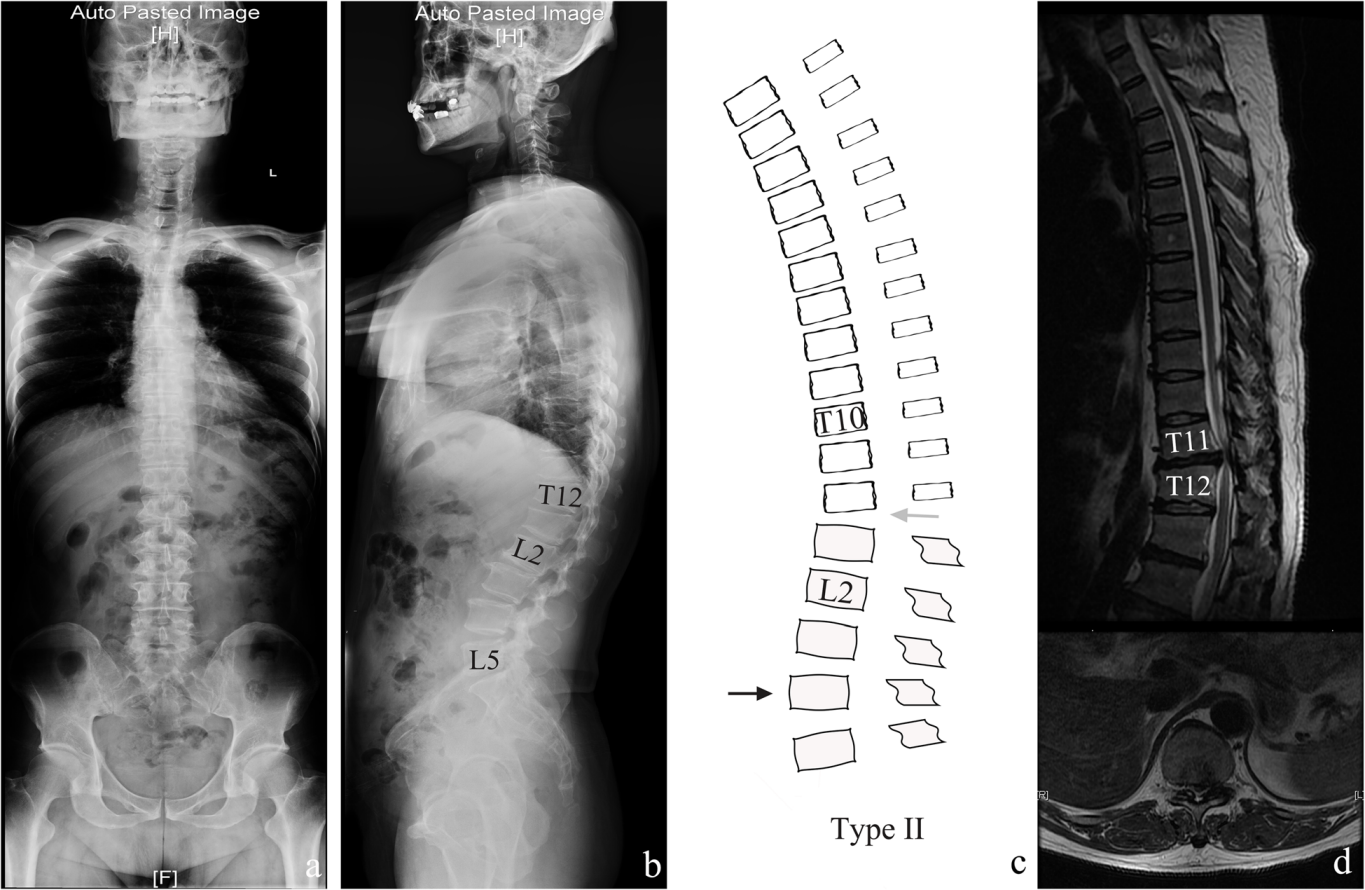
A 50-year-old man classified as type II. **a** Anterior-posterior whole spine x-ray demonstrated a balanced spine in coronal alignment. **b** Apex of lumbar lordic curve, apex of thoracic kyphotic curve, and inflexion point of two curves was identified as L5, T11/T12, and L2, respectively. A balanced spine in sagittal alignment was also observed in lateral x-ray. **c** Illustration of type II, and it had good agreement to a real case. **d** MR demonstrated disc herniation at T11/T12 level, and a TLDH-A was identified

We summarised each thoracic-lumbar alignment figure and found two subtypes differentiated by the apex of kyphotic curves (Fig. 1). Type I was similar to a normal spine curvature, defined as an apex of the thoracic kyphotic curve located in the middle thoracic spine, indicating that the whole thoracolumbar region was still below the apex of the thoracic kyphosis (Fig. 2). Type II was defined as an apex of the thoracic kyphotic curve located in the thoracolumbar region, indicating that the middle thoracic region was replaced by part of the thoracolumbar region as the apex of the thoracic kyphosis (Fig. 3). Thirty-six patients were classified as type I, and 34 patients were classified as type II.

### Details and comparison of the two subtypes

In type I patients, the apex of the thoracic kyphotic curve was at T5 in five cases, T6 in 11 cases, T7 in 12 cases, and T8 in eight cases. The apex of the lumbar lordotic curve was at L3 in one case, L3/4 in three cases, L4 in 16 cases, L4/5 in 11 cases, and L5 in five cases. The inflexion point was at T12 in 11 cases, L1 in 17 cases, and L2 in eight cases.

In type II patients, the apex of the thoracic kyphotic curve was at T10 in four cases, T10/T11 in three cases, T11 in 10 cases, T11/T12 in nine cases, T12 in two cases, T12/L1 in four cases, and L1 in two cases. The apex of the lumbar lordotic curve was at L3/4 in one case, L4 in seven cases, L4/5 in 14 cases, and L5 in 12 cases. The inflexion point was T12 in two cases, L1 in 13 cases, L2 in 16 cases and L3 in three cases.

There was a statistically significant difference in TLK (*p* < 0.001) and LL (*p* = 0.033) between the two subtypes, but there were no differences for other variants (Table 2).

**Table 2:** Comparison of parameters between subtypes

Parameters	Type I	Type II	*p* value
Age (yr)	47.4 ± 13.7 (25–69)	43.7 ± 15.4 (19–73)	0.242
BMI	26.1 ± 4.4 (20.4–38.0)	26.9 ± 4.2 (19.9–35.2)	0.381
DM (Yes/No)	2/34	4/30	0.463
Smoking (Yes/No)	3/33	5/29	0.307
Alcohol (Yes/No)	4/32	4/30	0.877
SVA(mm)	25.1 ± 16.5 (−31.5–71.2)	30.9 ± 31.7 (−46.9–73.1)	0.738
CVA(mm)	7.5 ± 4.4 (2.1–21.1)	8.5 ± 6.2 (2.3–26.1)	0.892
TK(°)	28.9 ± 11.2 (0.8–53.9)	29.2 ± 13.5 (3.4–56.0)	0.796
TLK(°)	14.9 ± 7.9 (0.9–31.5)	29.1 ± 13.7 (3.4–57.0)	< 0.001**
LL(°)	51.4 ± 10.9 (24.4–68.6)	43.3 ± 17.5 (7.5–71.4)	0.033*
T1PA(°)	8.2 ± 6.1 (0.3–23.6)	8.3 ± 8.5 (0.4–43.3)	0.573
L1PA(°)	6.0 ± 4.7 (0.3–19.6)	6.4 ± 5.2 (0.2–19.1)	0.930
PI(°)	47.1 ± 7.7 (30.7–63.6)	44.8 ± 7.3 (32.5–60.2)	0.206
SS(°)	35.7 ± 6.8 (20.7–51.1)	33.3 ± 8.8 (14.1–53.1)	0.247
PT(°)	11.4 ± 9.3 (0.2–30.5)	11.4 ± 9.5(0.5–38.3)	0.507
PI-LL(°)	10.4 ± 8.1 (0.5–36.3)	14.5 ± 10.3 (0.6–44.9)	0.111

** *p*<0.01, * *p*<0.05

### Association between TLDH level and regional curvatures

In type I patients, TLDH-I was recognized in 23 patients, TLDH-A in 0 patients, and TLDH-O in 13 patients. In type II patients, TLDH-I was recognised in three patients, TLDH-A in 29 patients, and TLDH-O in two patients. The Fisher’s exact test showed that TLDH-I was more frequently observed in type I patients (*p* < 0.001), while TLDH-A was more frequently observed in type II patients (*p* < 0.001).

### Concordance with the Roussouly classification

The distribution of match/mismatch ratios of the Roussouly classification in each subtype are shown in Table 3. In type I and II patients, 58.3 and 73.5% were matched with the Roussouly classification, respectively. There was a statistically significant difference between the two subtypes (*p* < 0.001).

**Table 3:** Concordance of Roussouly classfication

Roussouly Classification	Match/Mismatch (Type I)	Match/Mismatch (Type II)	*p* value
I	5/0	9/3	–
II	4/7	10/4	–
III	12/4	7/0	–
IV	0/4	1/0	–
Total	21/15	27/7	< 0.001**

** *p*<0.01

## Discussion

TLDH is a relatively rare condition, which has been defined in previous studies as thoracic disc herniation or upper lumbar disc herniation, and little is known about its regional alignment and aetiology. In this study, we found two typical subtypes of thoracic-lumbar alignment in TLDH patients, classified according to the apex of the thoracic curve. Regional deformity, but balanced alignment, was validated in both profiles, as interpreted from radiographic parameters.

In previous studies, many attempts have been made to depict spine alignment for a better understanding of spine disease [11–13]. Bae et al. investigated the spine alignment in upper lumbar disc herniation, which was defined as symptomatic disc herniation at L1/2 and L2/3 [13]. One limitation of this study was the neglect of thoracolumbar region alignment. In addition, TLK angle and other parameters were not recorded. The TLK angle has been reported to be less than 10° in the normal population, and the severity of kyphotic deformity is defined as mild (10–25°), moderate (26–50°), and severe (more than 50°) [14]. In this study, a TLK angle higher than the normal population was observed in both subtypes, indicating a common sagittal profile with thoracolumbar kyphotic deformity. The large TLK angle was validated as a risk factor for upper LDH by Wang, and a relatively higher mean TLK angle (16.9 ± 0.4°) was also observed in the TLDH group compared to the LDH group (7.6 ± 5.2°) in a comparative study [3, 15]. Our conclusion was consistent with that of previous studies, and we speculated that disc degeneration may be accelerated by excessive mechanical stress generated by a high TLK angle, resulting in the occurrence of TLDH.

Some radiographic parameters are considered as important tools for evaluating the balance status of spine alignment. SVA is the most widely used, and a novel parameter, T1PA, has attracted more attention [8]. As there are no reports about spine balance status in patients with TLDH, we first attempted to evaluate the spine balance status using these values, and found that both could define a balanced spine. Furthermore, L1PA was also utilised as a practical tool to evaluate regional lumbar curvature alignment, and a similar conclusion was drawn—a balanced lumbar spine. A balanced spine was observed in both whole spine alignment and regional spine alignment, indicating the wide range of compensatory ability of the spine, even in mild to moderate regional deformities.

Although a regional kyphotic deformity, but balanced spine, was observed in both subtypes, some different characteristics between type I and type II were also observed. First, we found that patients classified as type II had a higher TLK angle and lower LL value compared to patients classified as type I. Secondly, TLDH-I was the major manifestation in type I patients, and TLDH-A was the major manifestation in type II patients.

We considered that TLDH was caused by its intrinsic curvatures in each sagittal profile. In type I patients, the sagittal profile was similar to that of the normal population, and the thoracolumbar region was identified as the lower arc of the thoracic kyphotic curve and inflexion point between the thoracic curve and lumbar curve. Although mechanical stress was still diverted by the aligned thoracic-lumbar curve in this type, we speculated that disc degeneration was accelerated by regional kyphosis in the thoracolumbar junction and eventually caused disc herniation, identified by TLDH-I. On the contrary, the middle thoracic spine was replaced by the thoracolumbar region as the apex region in type II patients, resulting in the reciprocal change in regional thoracolumbar curvature whereby the thoracolumbar region was altered to the top of the thoracic and lumbar alignment. In this profile, the aligned thoracic-lumbar curve was disrupted, resulting in that excessive mechanical stress was directly loaded at the top of the curve (thoracolumbar apex region) rather than being diverted by an arc as in a normal population or type I patients, presenting as TLDH-A.

The Roussouly classification was first established in a normal population, and its effectiveness in pathologic conditions remained ambiguous. Some authors have used it as a practical tool for lumbar degenerative disease treatment, and the shape of curvature and SS values are well-matched in these populations [4, 11, 12]. However, mismatch of shape and radiographic values was frequently observed in patients with spine deformities, and some authors have attempted to reconstruct spine alignment according to this classification [7, 16, 17]. We found mismatches between parameters and shape in both types, which were different to findings for degenerative lumbar disease in previous studies [4, 11, 12]. We speculated this was the result of regional deformities. As we described in this study, regional kyphosis should be taken into consideration for TLDH aetiology in this “pathogenic but compensatory” sagittal profile.

To compensate for the Roussouly classification, Sebaaly further added the “anteverted type” or “retroverted type” category using PT values [18], and some authors have also advocated that compensatory pelvic retroversion was the possible compensatory mechanism of thoracolumbar kyphotic deformity [19, 20]. We attempted to interpret thoracolumbar profiles using PT values and set a PT value of more than 25° as pelvic retroversion and less than 5° as pelvic anteversion. A neutral pelvic morphology was frequently observed in both subtypes, indicating that compensatory pelvic mechanisms were not certain in TLDH. However, further studies with large samples are needed to validate these corresponding compensatory mechanisms.

### Limitations

This study had several limitations. First, it was retrospective and conducted at a single centre, limiting the generalisability of the results. Secondly, as we did not investigate the evolution from normal alignment to pathogenic alignment, further studies are needed to confirm our results. Thirdly, genetic factors, trauma, and other factors were not considered in this study. Furthermore, the compensatory mechanisms of the limbs should also be taken into consideration in future studies.

## Conclusions

We presented two distinctive sagittal profiles in TLDH patients, and a regional kyphotic deformity with a balanced spine was validated in both subtypes. In type I patients, disc degeneration was accelerated by regional kyphosis in the thoracolumbar junction and eventually caused disc herniation. In type II patients, excessive mechanical stress was directly loaded at the top of the curve (thoracolumbar apex region) rather than being diverted by an arc as in a normal population or type I patients. Mismatch between shape and sacral slope value was observed, and better agreement was found in Type II patients.

## Data Availability

The datasets used and/or analyzed during the current study available from the corresponding author on reasonable request.
